# Supplementary material for: The biomechanical origin of extreme wing allometry in hummingbirds
Source: Nat Commun. 2017 Oct 19;8:1047. doi: 10.1038/s41467-017-01223-x (PMC5715027; doi:10.1038/s41467-017-01223-x)
Supplement: Supplementary file 1 — Supplementary Information [file 41467_2017_1223_MOESM1_ESM.pdf]

SUPPLEMENTARY NOTE 1: SPECIES NAMING

We made the following decisions on species naming and phylogenetic placement:

1. It is not established that *Eriocnemis sapphiropygia* is distinct from *E. luciani,* so we use the *E. luciani* position in the tree for analysing *E. sapphiropygia* data.

2. It is not established that *Threnetes niger* is distinct from *T. leucurus,* so we use the *T. leucurus* position for *T. niger*.

3. *Haplophaedia assimilis* is not present in the phylogeny but is a recent split from *H. aeureliae,* and we therefore use the position of *H. aureliae*.

4. The data for *Eugenes fulgens* is obtained from the *E. fulgens fulgens* subspecies, but the phylogenetic placement on the tree is for *E. fulgens spectabilis*. However, because they are certainly sister taxa, we use the tip position of *E. f. spectabilis*.

5. The phylogenetic hypothesis suggests that the two subspecies of *Amazilia saucerrottei, A. s. hoffmanni* and *A. s. saucerrottei,* are actually distinct species; our single specimen is of *A. s. hoffmanni*.

6. Specimens labelled *Acestrura mulstant* were renamed *Chaetocercus mulsant* to match the current phylogenetic hypothesis.

7. Specimens labelled *Leucippus chionogaster* were renamed *Amazila chionogaster* to match the current phylogenetic hypothesis.

8. Specimens labelled *Saucerottia edward* were renamed *Amazilia edward* to match the current phylogenetic hypothesis.

9. *Chlorestes notatus* can take two possible positions on the tree[1] but is likely sister to *Damophila julie*. This uncertainty is included in the analysis by including both positions in the posterior distribution of the phylogenetic hypothesis.

SUPPLEMENTARY DISCUSSION: SCALING OF AERODYNAMIC AND INERTIAL POWER

Detailed experimental measurements of the drag on the wing are needed to estimate profile power, which was not possible in these field studies. Absent such studies, we must adopt the quasi-steady assumption of a nearly flat plate at low angles of attack, as in Ellington[2] in which case profile power is dominated by surface friction. This results in a dependence on the stroke-averaged Reynolds number (mean $Re=4\Phi R^2 f/\mu AR$, where μ is the kinematic viscosity calculated with Sutherland's formula[3] and AR is the aspect ratio), giving a profile drag coefficient[2] of $\bar{C}_{\mathrm{D,pro}} \approx 7/\sqrt{Re}$. From the exponents in Supplementary Table 1, Re approximately scales as $W^{0.5}$ among and within species, and the profile drag coefficient constructed this way would decline as $W^{-0.25}$. Summing allometric exponents of the profile power, $P_{\mathrm{pro}}=F_{\mathrm{pro}}\cdot\bar{U}= ½\rho S\bar{U}_3{}^3\bar{C}_{\mathrm{D,pro}}$, we would therefore predict that the specific profile power during hovering declines among, but not within, species (using allometric slopes in Supplementary Table 1).

The previous calculation of profile power is limited in two respects. First, Reynolds number variation does not significantly explain aerodynamic performance of spinning, prepared hummingbird wings[3,4] and so it is doubtful that Reynolds number is an appropriate predictor of profile drag coefficient variation among and within species. Aerodynamic performance is instead dominated by the Rossby number[5,6] the aspect ratio with respect to the center of rotation. Because aspect ratio does not vary with species body mass among species (Supplementary Table 1), it is similarly unlikely that the profile power drag coefficient varies among species. It may, though, increase somewhat within species due to small changes in aspect ratio. Reexamining the scaling of profile power with a constant $\bar{C}_{\mathrm{D,pro}}$, we predict that among species, $P_{\mathrm{pro}}^{*}\propto W^0$ because $S\propto W^1$ and $\bar{U}^3\propto W^0$. Conversely, within species $S\propto W^{0.41}$ and $\bar{U}^3\propto W^{0.75}$ which predicts $P_{\mathrm{pro}}^{*}\propto W^{0.16}$, and we would conclude that profile power increases within species more rapidly than among species (possibly compounded by increasing aspect ratio within species, Supplementary Table 1). We nonetheless emphasise that this method and Ellington's method (Supplementary Table 1) both predict that the scaling exponent of profile power is greater within species than among species. Because this is due to differences in the allometry of wing area and wing velocity, the general predictions of equation (4) are supported in both cases.

The cost of flight might also be influenced by the inertial power required to accelerate the wing at each stroke, which might increase with larger wing sizes. The contribution of inertial power to total power is unclear because of uncertainty in the magnitude of elastic energy storage. To estimate inertial power, we require knowledge of the total wing mass, m_{w}, and the wing mass moment of inertia, $\hat{r}_2(\mathrm{m})$. For ethical reasons, the wing mass and

moment were not collected for every individual but only obtained after incidental deaths during field experiments. We collected 26 measurements of the wing mass moments paired with wing areas from 10 species[7,8] and a further 10 mass moment measurements unpaired with wing area from one species[9]. Total wing mass, m_w, was very strongly correlated with wing area, body mass, and wing length across all individuals (correlation with wing area, r=0.968; m_w=-0.16 $S^{1.25}$, see also Supplementary Table 1). The wing mass was therefore imputed for all missing individuals from the predicted values of (non-phylogenetic) linear regression on wing area, body mass, and wing length, implemented in the R package *mice*[10]. The wing mass moment was not obviously correlated with any other parameter, suggesting it may be generally invariant among hummingbirds, and was therefore imputed from the second moment of wing area, $\hat{r}_2(S)$, the wing length, and the wing area. Each reported imputed value is the mean of five multiple imputation chains.

Inertial power was then calculated according to the method of Ellington[2]. Specific inertial power requirements are constant among species (Supplementary Table 1), indicating that the increasing weight of the wing is offset by the decreasing frequency of accelerations (decrease in stroke frequency). Conversely, specific inertial power increases within species. Total power, assuming no elastic energy storage (P_{zero}^*), is independent of body mass among species, but increases with weight within species. We do not include inertial power in the main text because of the uncertainty in elastic energy storage, and because we have not been able to study the possibility that the relationship of wing mass and wing area differs among and within species. Assuming this relationship is equivalent, then the total power with no elastic energy storage (P_{zero}^*) is independent of body weight among species, but increases with weight within species (Supplementary Table 1). With the preceding caveats, the scaling of inertial power thus supports our arguments as well.

Supplementary Table 1 Table of sample sizes, exponents, and 95% credible intervals for variables in this study.

Variable	Symbol	N	n	Interspecific (2.5%,97.5%)	Intraspecific (2.5%,97.5%)
Air density	ρ	112	1446	-0.058 (-0.112,-0.003)	-0.065 (-0.085,-0.045)
Wing length	R	112	1433	0.498 (0.455,0.539)	0.228 (0.201,0.256)
Wing length corrected for $\hat{r}_2$	R_2	89	901	0.491 (0.441,0.539)	0.223 (0.185,0.261)
Non-dimensional second moment of area	$\hat{r}_2$	89	900	-0.008 (-0.016,0.001)	0.006 (-0.003,0.014)
Non-dimensional third moment of area	$\hat{r}_3$	89	893	-0.011 (-0.021,0.000)	0.010 (-0.002,0.021)
Wing surface area	S	112	1432	1.012 (0.908,1.113)	0.417 (0.366,0.468)
Wing aspect ratio	AR	112	1431	-0.023 (-0.062,0.017)	0.039 (0.006,0.073)
Wing velocity	$\bar{U}$	84	593	0.010 (-0.054,0.074)	0.268 (0.182,0.354)
Wing velocity (burst)	$\bar{U}_\mathrm{b}$	81	571	0.037 (-0.006,0.081)	0.190 (0.121,0.258)
Wing tip velocity	$\bar{v}_\mathrm{tip}$	84	607	0.018 (-0.046,0.082)	0.249 (0.165,0.333)
Wing tip velocity (burst)	$\bar{v}_\mathrm{b,tip}$	81	585	0.053 (0.010,0.096)	0.180 (0.113,0.248)
Force coefficient	$\bar{C}_\mathrm{w,V}$	84	593	0.007 (-0.122,0.137)	0.097 (-0.094,0.289)
Force coefficient (burst)	$\bar{C}_\mathrm{b,V}$	81	581	-0.059 (-0.147,0.029)	0.057 (-0.111,0.223)
Stroke frequency	f	84	610	-0.474 (-0.557,-0.39)	-0.039 (-0.110,0.031)
Stroke frequency (burst)	f_b	81	604	-0.428 (-0.496,-0.358)	-0.050 (-0.103,0.002)
Stroke amplitude	Φ	84	607	0.005 (-0.038,0.046)	0.049 (-0.013,0.110)
Stroke amplitude (burst)	Φ_b	81	585	0.001 (-0.023,0.024)	-0.003 (-0.033,0.033)
Total lifted mass	M_T	81	623	0.987 (0.889,1.084)	0.759 (0.632,0.887)
Load factor	n	81	623	-0.013 (-0.112,0.082)	-0.235 (-0.364,-0.107)
Wing mass†	w_m	15	33	1.375 (0.996,1.705)	-
Induced power	$P_\mathrm{w,ind}^*$	84	607	0.015 (-0.033,0.063)	0.254 (0.193,0.315)
Induced power (burst)	$P_\mathrm{b,ind}^*$	81	581	0.067 (-0.21,0.15)	-0.032 (-0.212,0.147)
Profile power	$P_\mathrm{w,pro}^*$	84	593	-0.269 (-0.413,-0.123)	-0.059 (-0.283,0.166)
Profile power (burst)	$P_\mathrm{b,pro}^*$	81	571	-0.182 (-0.307,-0.057)	-0.254 (-0.460,-0.048)
Inertial power	$P_\mathrm{w,acc}^*$	76	535	0.063 (-0.104,0.237)	0.408 (0.16,0.655)
Inertial power (burst)	$P_\mathrm{b,acc}^*$	76	529	0.150 (0.034,0.268)	0.233 (0.023,0.443)
Total power (Ellington) – perfect	$P_\mathrm{w,per}^*$	84	587	-0.033 (-0.074,0.009)	0.19 (0.145,0.235)
Total power (Ellington) (burst) – perfect	$P_\mathrm{b,per}^*$	81	565	0.036 (-0.069,0.137)	-0.058 (-0.220,0.107)
Total power (Ellington) – zero	$P_\mathrm{w,zero}^*$	76	529	0.05 (-0.086,0.188)	0.352 (0.153,0.550)
Total power (Ellington) (burst) – zero	$P_\mathrm{b,zero}^*$	76	523	0.119 (0.025,0.212)	0.138 (-0.037,0.313)

† calculated using MCMCglmm

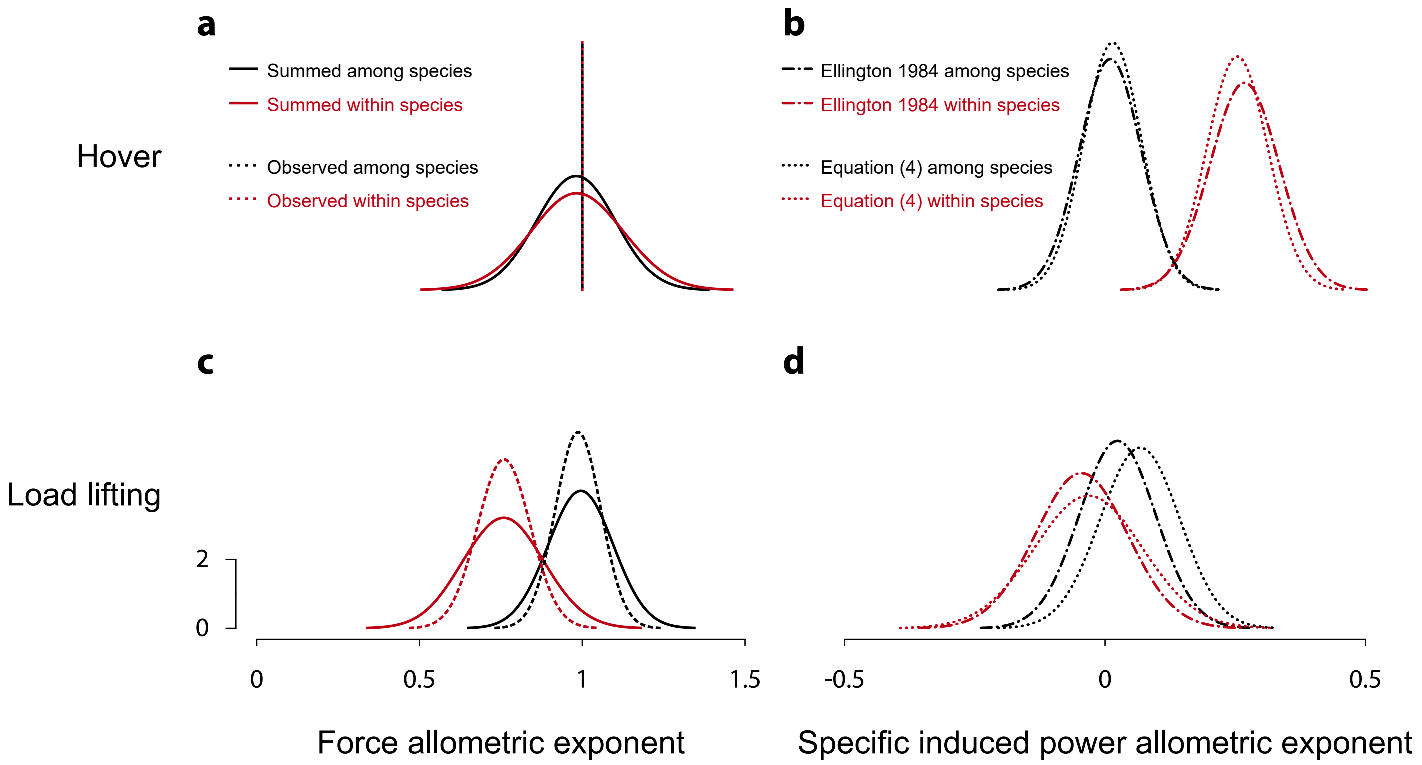

Supplementary Figure 1 Comparison of different methods of reconstructing the allometry of force (**a**,**b**) and specific induced power (**c**,**d**). **a** In hovering, the force produced is exactly equal to body weight, which we therefore 'observe' to be exactly equal to 1 (vertical dashed lines) both among and within species. For predictions derived from equation (2) to be valid for hovering flight, it is necessary that the sum of the posterior distributions of each term (solid lines) match the observed force generation, and must therefore be centered on 1. This condition is met both among (black) and within (red) species. **b** During burst performance, the allometry of force generation may differ from unity. The exponent of the empirically measured burst force (dashed lines) is compared to the reconstructed burst force obtained by summing the exponents of each term as measured during the assay (solid lines). Among and within species, the two methods again substantially agree with each other. **c**,**d** In this study, the allometry of specific induced power cannot be observed directly, but must be computed either as described by Ellington (1984)[2] and in the Supplementary Methods (long-dashed lines), or by summing the contributions of each component in equation (4) (dotted lines). Specific induced power exhibits significant positive allometry in hovering (**c**) within species, but neither among nor within species during load lifting (**d**). Distributions are smoothed with bandwidth=0.05.

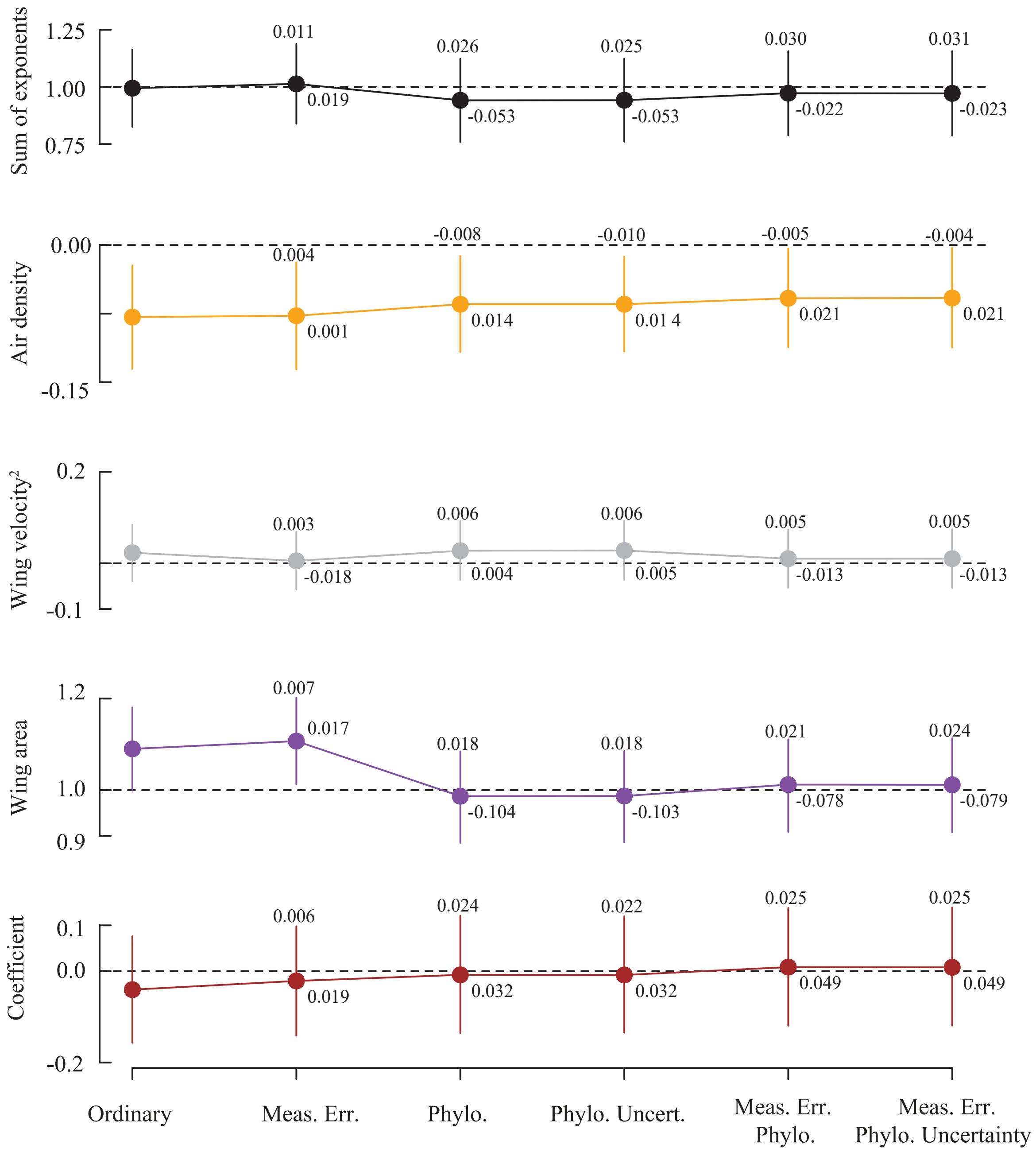

Supplementary Figure 2 Effect of modelling assumptions on estimated allometric exponents and sum of exponents. We performed regressions using ordinary least squares (Ordinary), and for combinations of accounting for intraspecific variation (Meas. Err.), including a phylogenetic hypothesis (Phylo.), and accounting for uncertainty in the phylogenetic hypothesis (Phylo. Uncert.). The difference in the mean and width of credible intervals are printed next to the circle (mean) or upper range of credible intervals. Effects were unpredictable and variable-specific, but in no case did we find that accounting for phylogenetic uncertainty contributed any more than accounting for the maximum clade credibility phylogenetic hypothesis alone. Air density was influenced by phylogenetic relatedness and its interaction with measurement errors; wing tip velocity was primarily influenced by measurement errors; wing area was greatly influenced by phylogenetic relatedness, but there were interactions between the phylogenetic hypothesis and measurement error; the force coefficient was influenced by both measurement error and phylogenetic relatedness.

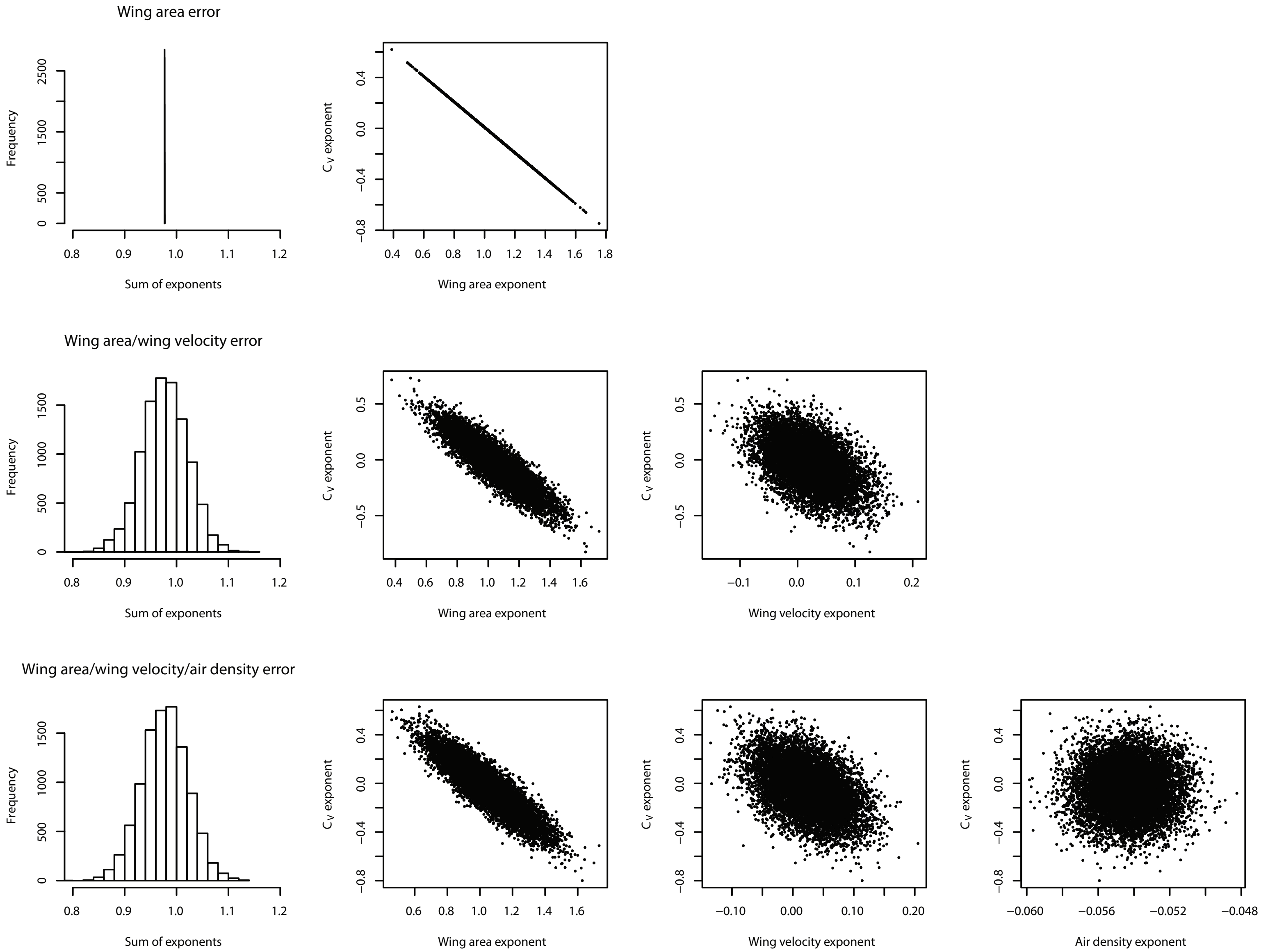

Supplementary Figure 3 Effect of force coefficient calculation on sum-to-one constraint. We simulate how the calculation of the force coefficient, C_V, from the empirical observations affects the sum-to-one constraint, and whether the constraint is trivially true. We use the empirical species mean of body mass, wing area, wing velocity, and air density. For each simulation, we add random error to the species mean of one or more variables (Gaussian error with standard deviation equal to 0.1 of each variable's mean), recalculate C_V for each species with these new values, and then repeat our scaling analysis. In row 1, we show that when only a single variable, wing surface area, contains error, the error in C_V is simply equal to the error in surface area. In this case, the sum of exponents is always equal to the true (empirical) sum, as shown in the histogram, showing that indeed, the constraint is trivial. When more than one variable has error (rows 2-3), as in any real system, the sum of exponents in any given experiment is not equal to the empirical sum. The width of the resulting distribution, i.e., the range of possible apparent force allometries that can be obtained, depends on the magnitude of errors in each variable (simulations not shown).

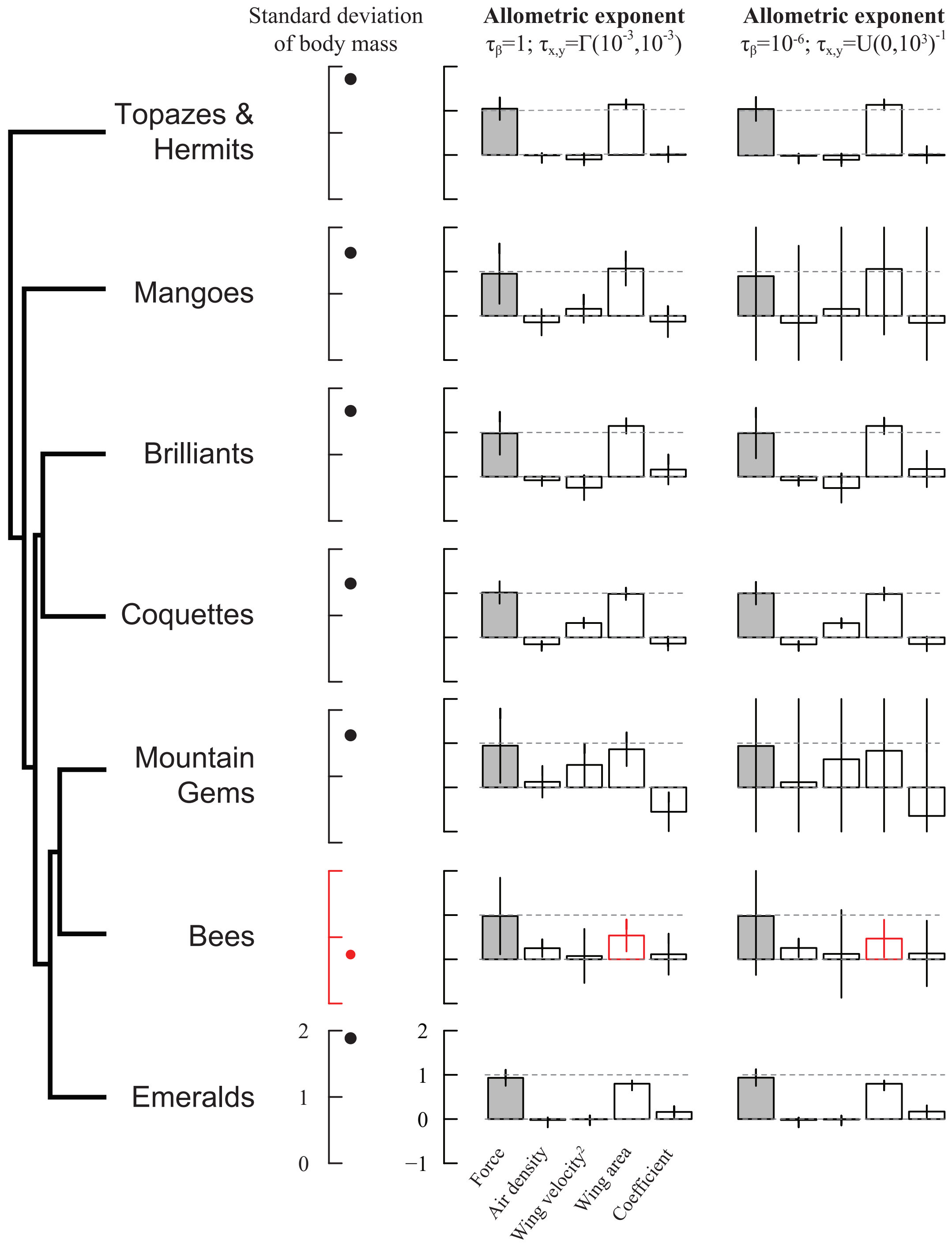

Supplementary Figure 4 Force allometry and mass variation in individual hummingbird clades. Comparisons within clades were made using the flat priors for precision ($\tau=\sigma^{-2}$) used for the general modelling, which resulted in very large posterior variances (error bars, clipped at borders). Prior distributions are discussed in Methods. We also examined trends using informative priors focused on $\beta=1$ for wing area, and $\beta=0$ for all other parameters. The general among and within-species patterns are visible in most clades: weight support among species is derived primarily from large wing area exponents. A possible exception is the Bee clade, which has exceptionally low wing area scaling, and also comparatively low intraclade variation in body mass. Topazes were combined with hermits due to insufficient sample size.

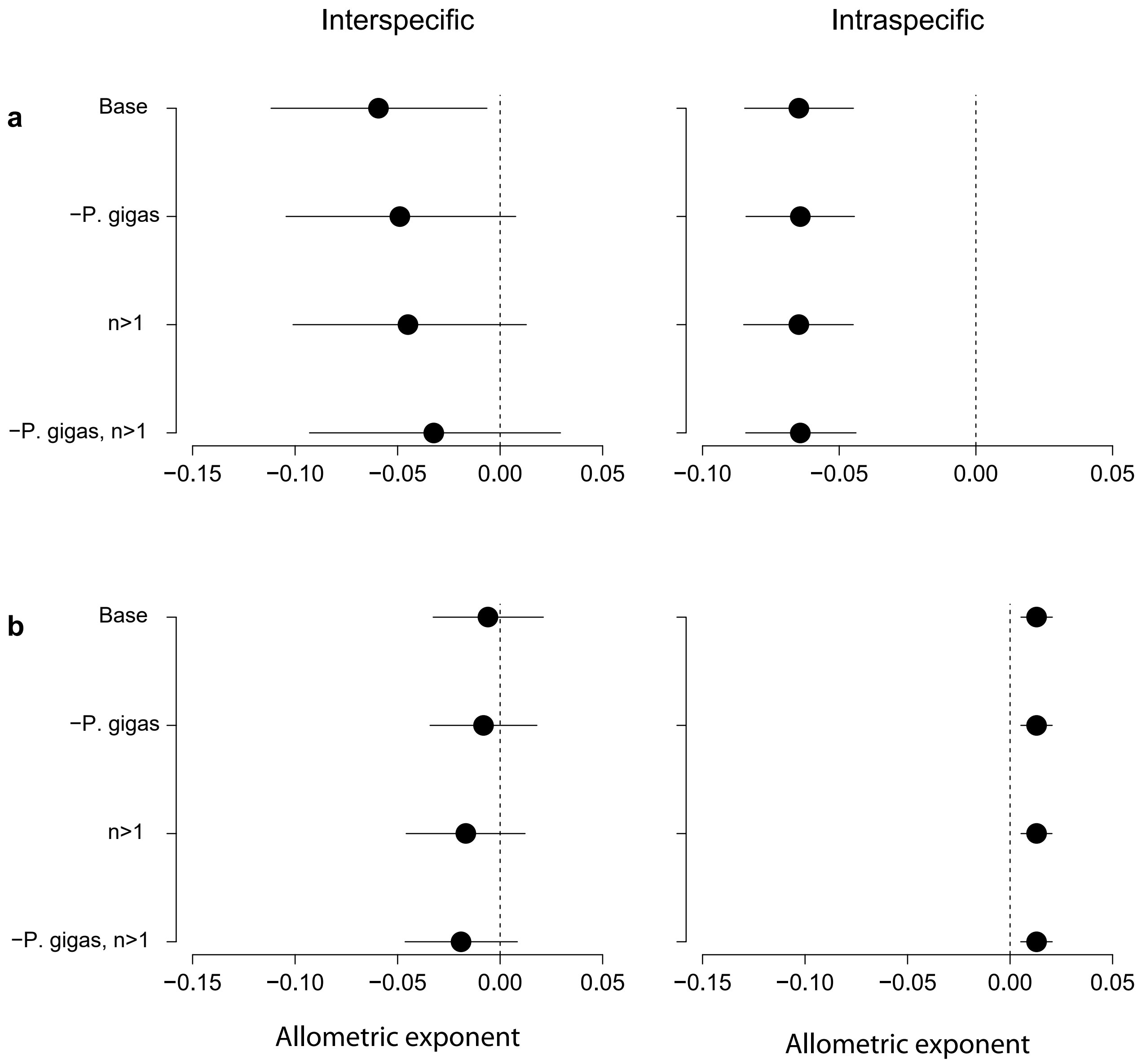

Supplementary Figure 5 Impact of data subsets on the estimated slope of air density on body weight. **a** Effect of removing *Patagona gigas* and poorly sampled species on estimated air density regression exponents. Regression on the complete data set suggests a small but significant contribution of air density to force allometry across hummingbirds. However, performing regressions after filtering out P. gigas (-P. gigas) or species with a single observation (*n*>1), or both, results in progressive diminuation of the exponent toward zero, suggesting either that this is not a robust biological observation or that we do have sufficient evidence to resolve such a relationship. Subsetting did not alter the trend within species. **b** To compare with previous work[7,11] we also examine the regression exponent of body mass as a function of elevation directly. All slopes among species overlap zero, whereas all slopes within species do not. Exponents were estimated with MCMC-glmm, and may therefore differ from models implemented in JAGS (see Materials and Methods).

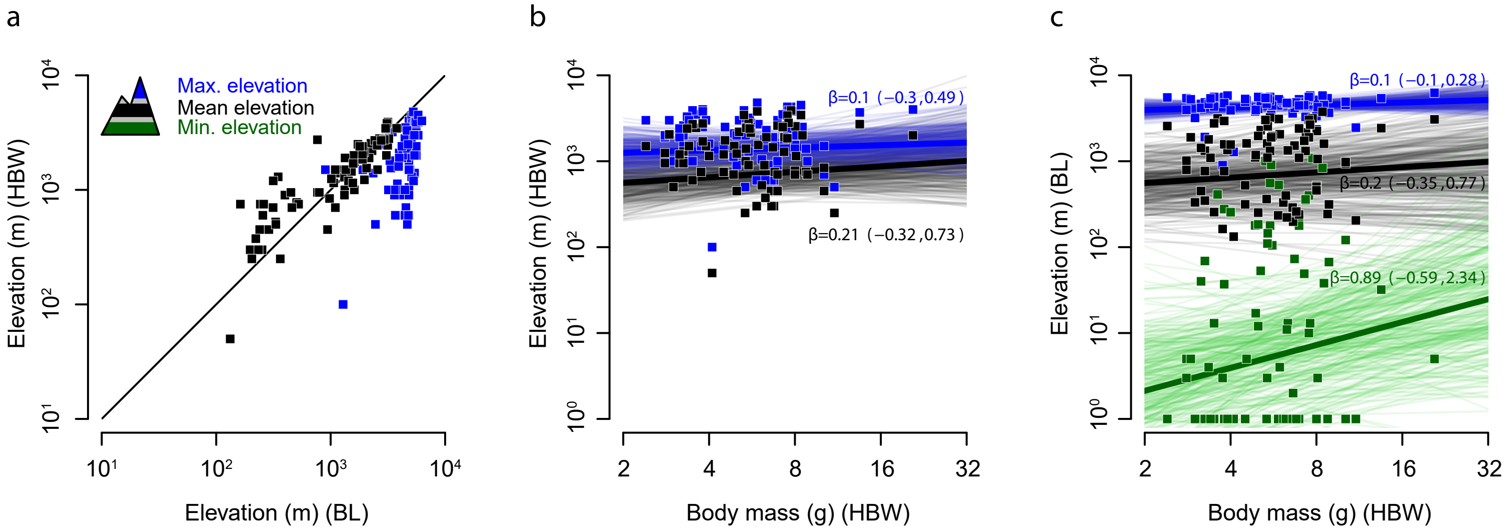

Supplementary Figure 6 No association between literature-derived species mean body mass and elevation. To support our conclusion that a linear change in body mass with elevation or air density is not a general feature of hummingbird biology, we collected an independent data set of species mean body masses and elevations. Mean species mass and mean and maximum elevation were obtained from the literature (HBW), and minimum, mean, and maximum elevations were also estimated from species range maps (BL). Mean elevations obtained from both methods were reasonably well correlated, but maximum elevations diverged substantially (**a**). We do not find any association between body mass and elevation (**b**,**c**).

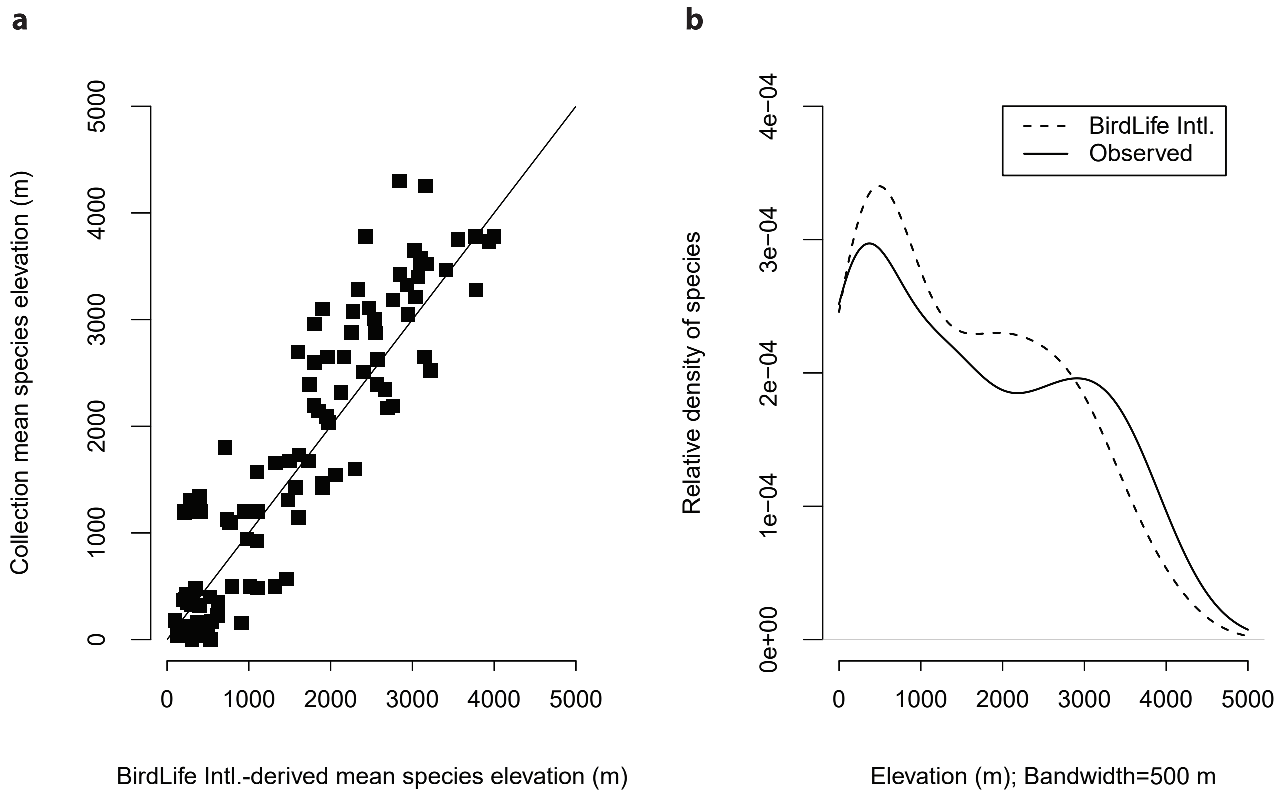

Supplementary Figure 7 Concordance between sampled and theoretical species mean elevations. The species mean elevations calculated from our sampling and those derived from digital range maps (**a**) were strongly correlated. Our elevational sampling also broadly conforms to the overall distribution of species mean elevations (**b**).

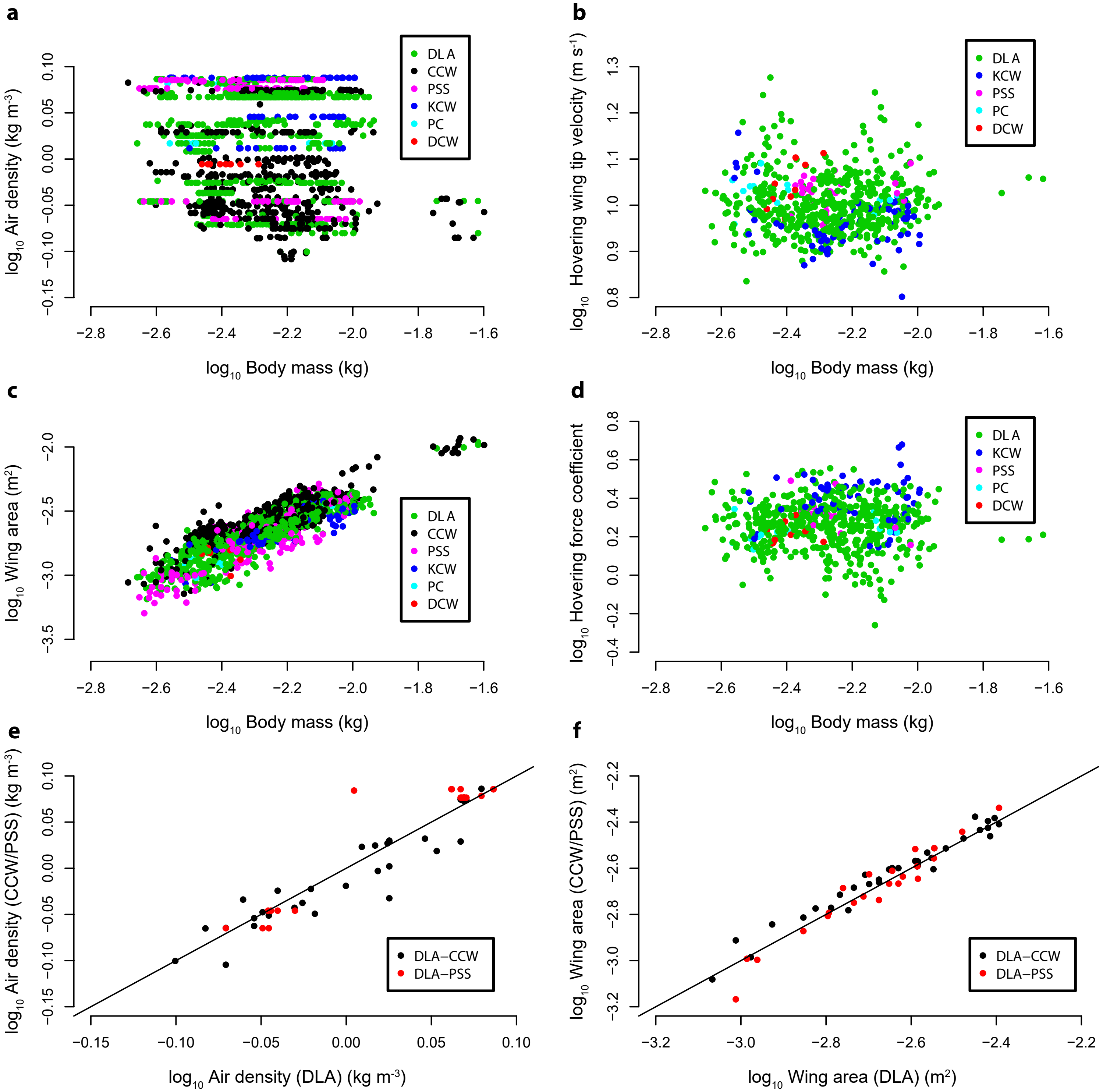

Supplementary Figure 8 Comparison of collector data sets for systematic bias. Each force allometric component (**a-d**) was examined for systematic biases. Where species observations overlapped, we compared the estimated trait mean (**e**,**f**). Species means among authors were in good concordance, suggesting that apparent differences among authors are most likely due to species sampling.

SUPPLEMENTARY REFERENCES

1. McGuire, J. A. *et al.* Molecular phylogenetics and the diversification of hummingbirds. *Curr. Biol.* (2014). doi:10.1016/j.cub.2014.03.016
2. Ellington, C. P. The aerodynamics of hovering insect flight. VI. Lift and power requirements. *Philos. Trans. R. Soc. Lond. B Biol. Sci.* **305,** 145–181 (1984).
3. Kruyt, J. W., Quicazán-Rubio, E. M., Heijst, G. F. van, Altshuler, D. L. & Lentink, D. Hummingbird wing efficacy depends on aspect ratio and compares with helicopter rotors. *J. R. Soc. Interface* **11,** 20140585 (2014).
4. Kruyt, J. W., Heijst, G. F. van, Altshuler, D. L. & Lentink, D. Power reduction and the radial limit of stall delay in revolving wings of different aspect ratio. *J. R. Soc. Interface* **12,** 20150051 (2015).
5. Lentink, D. & Dickinson, M. H. Biofluiddynamic scaling of flapping, spinning and translating fins and wings. *J. Exp. Biol.* **212,** 2691–2704 (2009).
6. Phillips, N., Knowles, K. & Bomphrey, R. J. The effect of aspect ratio on the leading-edge vortex over an insect-like flapping wing. *Bioinspir. Biomim.* **10,** 056020 (2015).
7. Altshuler, D. L., Dudley, R., Heredia, S. M. & McGuire, J. A. Allometry of hummingbird lifting performance. *J. Exp. Biol.* **213,** 725–734 (2010).
8. Wells, D. J. Muscle performance in hovering hummingbirds. *J. Exp. Biol.* **178,** 39–57 (1993).
9. Chai, P. & Dudley, R. Limits to flight energetics of hummingbirds hovering in hypodense and hypoxic gas mixtures. *J. Exp. Biol.* **199,** 2285–2295 (1996).
10. van Buuren, S. & Groothuis-Oudshoorn, K. Multivariate imputation by chained equations in R. *J. Stat. Softw.* **45,** 1–67 (2011).
11. Altshuler, D. L., Dudley, R. & McGuire, J. A. Resolution of a paradox: Hummingbird flight at high elevation does not come without a cost. *Proc. Natl. Acad. Sci. U. S. A.* **101,** 17731–17736 (2004).
